# Correlation of *rpoB* Mutations with Minimal Inhibitory Concentration of Rifampin and Rifabutin in *Mycobacterium tuberculosis* in an HIV/AIDS Endemic Setting, South Africa

**DOI:** 10.3389/fmicb.2016.01947

**Published:** 2016-12-05

**Authors:** Ivy Rukasha, Halima M. Said, Shaheed V. Omar, Hendrik Koornhof, Andries W. Dreyer, Alfred Musekiwa, Harry Moultrie, Anwar A. Hoosen, Gilla Kaplan, Dorothy Fallows, Nazir Ismail

**Affiliations:** ^1^Department of Medical Microbiology, Faculty of Health Sciences, University of the Free StateBloemfontein, South Africa; ^2^Centre for Tuberculosis, National Institute for Communicable DiseasesSandringham, South Africa; ^3^Department of Medical Microbiology, Faculty of Health Sciences, University of the WitwatersrandJohannesburg, South Africa; ^4^Centre for Evidence-based Health Care, Faculty of Medicine and Health Sciences, Stellenbosch UniversityCape Town, South Africa; ^5^Medical Research Council, Respiratory and Meningeal Pathogens Research Unit, School of Pathology, University of the WitwatersrandJohannesburg, South Africa; ^6^The Bill and Melinda Gates Foundation, SeattleWA, USA; ^7^Public Health Research Institute, New Jersey Medical School, The State University of New Jersey, Rutgers University, NewarkNJ, USA; ^8^Department of Medical Microbiology, Faculty of Health Sciences, University of PretoriaPretoria, South Africa

**Keywords:** *Mycobacterium tuberculosis*, *rpo*B, rifampicin, rifabutin, minimum inhibitory concentration, HIV/AIDS, South Africa, point mutation

## Abstract

Treatment of tuberculosis (TB) and HIV co-infections is often complicated by drug-to-drug interactions between anti-mycobacterial and anti-retroviral agents. Rifabutin (RFB) is an alternative to rifampin (RIF) for TB regimens and is recommended for HIV patients concurrently receiving protease inhibitors because of reduced induction of CYP3A4. This study sought to determine the proportion of RFB susceptible isolates among RIF-resistant strains in a high HIV prevalence setting in South Africa. In addition, the study explored the association between *rpoB* mutations and minimum inhibitory concentrations (MIC) of RIF and RFB. A total of 189 multidrug resistant (MDR) *Mycobacterium tuberculosis* isolates from the Centre for Tuberculosis repository were analyzed. The MICs were determined using a MYCOTB Sensititre plate method and the *rpoB* gene was sequenced. Of the 189 MDR isolates, 138 (73%) showed resistance to both RIF and RFB, while 51 (27%) isolates were resistant to RIF but retained susceptibility to RFB. The S531L was the most frequent *rpoB* point mutation in 105/189 (56%) isolates, followed by H526Y in 27/189 (14%) isolates. Resistance to both RIF and RFB was found predominantly in association with mutations S531L (91/105, 87%), H526Y (20/27, 74%), and H526D (15/19, 79%), while D516V (15/17, 88%), and L533P (3/4, 75%) were found in RIF-resistant, RFB-susceptible isolates. This study has shown that up to 27% of MDR-TB patients in South Africa may benefit from a treatment regimen that includes RFB.

## Introduction

Tuberculosis (TB) is responsible for 25% of HIV/AIDS related mortality worldwide, with sub-Saharan Africa accounting for 79% of HIV-associated TB cases (WHO, 2015). In South Africa, 65% of TB patients are HIV-positive, and TB remains the leading cause of death among HIV-infected individuals. Treatment of TB in the context of HIV co-infection is challenging, due to the high potential for drug-drug interactions in combined antimicrobial and anti-retroviral (ARV) chemotherapy. There is an urgent need to harmonize TB and HIV treatment through development of compatible ARV regimens. Moreover, multidrug-resistant (MDR) TB, defined by resistance to rifampin (RIF) and isoniazid (INH), is emerging, particularly within high burden countries, such as South Africa (WHO, 2015). MDR-TB is associated with poor treatment outcomes and greatly elevated health costs.

Due to its sterilizing capacity, the inclusion of RIF in TB treatment regimens is crucial for achievement of high cure rates coupled with low relapse rates (CDC, 2013). However, RIF is a potent inducer of CYP3A4 and other cytochrome P450 enzymes, leading to reduced serum levels of protease inhibitors, used in treatment of HIV/AIDS (CDC, 2013). Rifabutin (RFB) is an alternative rifamycin, which has less impact on CYP3A4 activity and improved pharmacokinetics compared to RIF (Regazzi et al., 2014). Although the activity of RFB is comparable to that of RIF for treatment of drug-susceptible TB, current guidelines recommend limited use of RFB only in drug-susceptible adult TB patients with HIV/AIDS or adults experiencing intolerance to RIF. Widespread use of RFB has also been limited by its cost and absence from most commercial susceptibility testing systems (Horne et al., 2011). However, RFB costs have been lowered by its addition to the WHO Essential Medicines List, while the recently validated MYCOTB Sensititre plate method includes RFB in its drug panel (Lee et al., 2014).

Resistance to both RIF and RFB is largely associated with mutations in an 81-bp RIF resistance determining region (RRDR) within the *rpoB* gene of *Mycobacterium tuberculosis* (Jamieson et al., 2014). Although high-level cross-resistance between the two rifamycins is reported, some studies have shown RFB susceptibility in RIF-resistant strains of *M. tuberculosis* in association with specific *rpoB* mutations (Cavusoglu et al., 2004; Yoshida et al., 2010; Jamieson et al., 2014; ElMaraachli et al., 2015). Thus, it has been argued that knowing the type of *rpoB* mutation may have clinical implications for guiding rifamycin-based therapeutic regimens (Sirgel et al., 2013; Berrada et al., 2016).

Studies from low HIV settings have reported that 13–26% of MDR-TB isolates show sensitivity to RFB (Chen et al., 2012; Jo et al., 2013; Schon et al., 2013). However, there is limited information on the frequency of RFB susceptibility among MDR-TB isolates in an HIV endemic region. This study aimed to determine the proportion of MDR strains with RFB susceptibility in Gauteng Province, South Africa. Gauteng is the economic hub of South Africa, with a large migrant workforce, where 73% of TB patients are co-infected with HIV. In addition, we examined correlations between specific *rpoB* mutations and the minimum inhibitory concentrations (MIC) of RIF and RFB among clinical MDR-TB isolates.

## Materials and Methods

### Clinical Isolates and Ethics

A total of 211 MDR-TB isolates available from the Centre for Tuberculosis (CTB) repository were included. These isolates were collected over the first 6 months of 2010 at the National Health Laboratory Services (NHLS) Central TB diagnostics laboratory in Braamfontein, Johannesburg from confirmed MDR-TB cases and submitted for analysis to the CTB, as described (Said et al., 2016).

Ethics approval for this study was obtained from the Research Ethics Committee of the Faculty of Health Sciences, University of the Free State (Ref: 230408-011).

### Minimal Inhibitory Concentration Determination of *M. tuberculosis* Isolates

Minimum inhibitory concentrations were determined using a commercially available Sensititre MYCOTB plate (TREK Diagnostics, Cleveland, OH, USA), following the manufacturer’s instructions. The MIC test range for both RIF and RFB was from 0.12 to 16 mg/L. Resistance and sensitivity to RIF were defined as MIC > 1 and MIC ≤ 1 mg/L, respectively, and to RFB as MIC > 0.5 and MIC ≤ 0.5 mg/L, respectively, based on laboratory standards (CLSI, 2011).

### DNA Extraction, PCR, and Sanger Sequencing

All isolates were grown on Löwenstein–Jensen agar; genomic DNA was extracted using the phenol-chloroform (CTAB) method (van Embden et al., 1993). Six primer sets were used for PCR amplification of the entire *rpoB* gene (**Table 1**). The PCR amplification protocol consisted of a 5 min denaturation step at 95°C, followed by 35 cycles of 30 s at 95°C, 30 s at 62°C and 50 s at 72°C and a final extension step at 72°C for 2 min. Following Sanger sequencing of amplicons, mutations in *rpoB* were identified by alignment to H37Rv reference strain (NCBI Accession number AL123456; Cole, 2002) using ClustalW2 (Li et al., 2015).

**Table 1:** *rpoB* primers used to amplify RRDR region.

Primer set	Forward primers (5′-3′)	Reverse primers (5′-3′>)	Amplicon size (nt)
rpoB-RRDR	GGGAGCGGATGACCACCCA	GCGGTACGGCGTTTCGATGAAC	350
rpoB-2	ATGACGTACGCGGCTCCACTGTTCG	GGTGGTCATCCGCTCCCGGACCAC	840
rpoB-3	CGCGGCGAACGGGCCCGTGGGCA	CGGGATCACCTTGACGCTGTGCAG	675
rpoB-4	CTGTCGGTGTACGCGCGGGTCAA	GGGACCGTCGGCGATCACCTGACC	621
rpoB-5	CCACGGCACTTGCGCCAACCAG	CATCCGTCGCGGCACGCCGTGGGT	742
rpoB-6	CCGGTTGAGGACATGCCGTTC	TCCCTTTCCCCTAACGGGTTTAGT	879

### Statistical Analysis

Kruskal–Wallis tests were used to determine whether mutations were associated with differences in RIF and RFB MICs. A Dunn test incorporating the Benjamini–Hochberg false discovery rate correction was performed to identify pairwise differences in RFB MICs.

## Results

Of the 211 MDR isolates in the collection, MIC and sequencing data were available for 189 (90%). The remaining 22 (10%) isolates were excluded from analysis due to RIF resistance not confirmed on MIC testing, contamination, or loss of viability.

Among the 189 MDR isolates analyzed, S531L was the most frequently observed *rpoB* RRDR mutation, found in 105/189 (56%) isolates, followed by H526Y in 27 (14%), H526D in 19 (10%), D516V in 17 (9%), L533P in four (2%), and D516G_L533P in three (2%) isolates (**Table 2**). Of the 189 isolates, 138 (73%) showed resistance to both RIF and RFB, while 51 (27%) were RIF-resistant but exhibited RFB susceptibility. Resistance to both RIF and RFB was predominantly associated with S531L (91/105, 87%), H526Y (20/27, 74%), and H526D (15/19, 79%) mutations. Rifabutin susceptibility was most commonly observed for isolates carrying D516V (15/17, 88%) and L533P (3/4, 75%), although two of three isolates with the double mutation D516G_L533P were moderately resistant to RFB. Nine (5%) RIF-resistant isolates had no mutations in the RRDR. However, three of the nine had a mutation outside the RRDR. Two of these (V276L and V276F) were RFB susceptible, while one (V252E) was RFB-resistant; the remaining six had no mutations in *rpoB* outside the RRDR.

**Table 2:** Mutations in *rpoB* RRDR and MICs of RIF and RFB for all MDR isolates.

Mutation	Sequence change	Total number of isolates	Number of RFB-S (% total)	MIC (ug/ml)		Number of isolates	Median MIC	
				RIF	RFB		RIF	RFB
S531L	TCG > TTG	105	14 (13)	16	16	4	16	2
				16	8	5		
				4	8	1		
				16	4	21		
				16	2	31		
				8	2	1		
				16	1	28		
				16	0.5	10		
				16	0.25	2		
				8	0.12	1		
				4	0.5	1		
H526Y	CAG > TAC	27	7 (26)	16	16	5	16	2
				16	8	2		
				16	4	3		
				16	2	6		
				16	1	4		
				16	0.5	5		
				16	0.25	2		
H526D	CAC > GAC	19	4 (21)	16	16	2	16	2
				16	8	4		
				16	4	2		
				16	2	5		
				16	1	2		
				16	0.5	4		
D516V	GAC > GTC	17	15 (88)	16	4	1	4	0.12
				16	1	1		
				16	0.25	1		
				8	0.25	1		
				2	0.25	1		
				16	0.12	1		
				8	0.12	3		
				4	0.12	5		
				2	0.12	3		
L533P	CTG > CCC	4	3 (75)	16	1	1	16	0.31
				16	0.5	1		
				16	0.12	2		
D516G_L533P	GAC_CTG > GGC_CCC	3	1 (33)	16	4	1	16	1
				16	1	1		
				16	0.5	1		
S531Q	TCG > CAG	1	0	16	2	1		
H526P	CAC > CCC	1	0	16	4	1		
H526R	CAC > CGC	1	0	16	4	1		
H526L	CAC > CTC	1	1	4	0.12	1		
Ins F at 514	Ins-3 bp TTC	1	1	8	0.5	1		
WT^∗^		9	5 (56)	16	4	1	16	0.5
				16	1	3		
				16	0.5	1		
				16	0.25	2		
				4	0.25	1		
				4	0.12	1		
Total						189		

*^∗^WT, wild type – no mutations in *rpo*B RRDR.*

**FIGURE 1:**
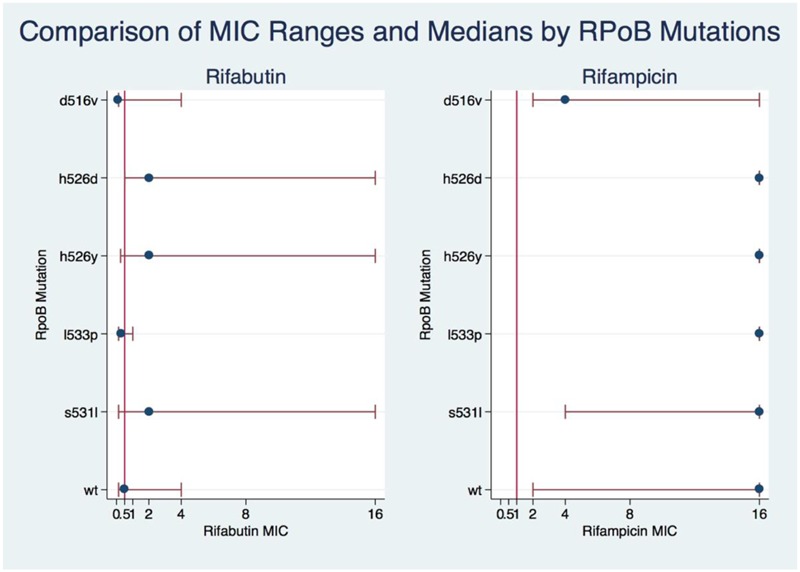
**Comparison of RIF and RIFB MIC ranges and medians by *rpoB* mutations.** A kruskal-Wallis test was used to show the association of MIC levels of RIF and RIFB with the *rpoB* mutations. The eclplot was constructed with Stata 14.

The Kruskal–Wallis test showed significant association between mutations and MICs for both RIF and RFB (Kruskal–Wallis statistic for RIF: *H* = 67.699, *p* = 0.0001; for RFB: *H* = 42.988, *p* = 0.0003). Dunn’s pairwise comparison with Benjamini–Hochberg false discovery rate correction showed that RFB MICs in D516V and L533P isolates were not significantly different from wild type (*q* = 0.1317), nor from each other (*q* = 0.4118), although the numbers were small (WT *n* = 9; L533P *n* = 4; D516V *n* = 17). The RFB MICs for H526D H526Y and S531L were not significantly different from each other. However, there were significant differences between RFB MICs in D516V isolates and those in H526D, H526Y, and S531L (all *q* < 0.0001). Among isolates with the most common RRDR mutations, there was a significantly lower median MIC for RFB compared to RIF, with at least a threefold lower median MIC for S531L, H526Y, and H526D; and 5- and 7-fold lower medians for D516V and L533P, respectively (**Figure 1**). Additionally, higher levels of resistance to RIF correlated with higher resistance to RFB (Spearman’s correlation coefficient *r* = 0.4511, *p* < 0.000001).

## Discussion

This study sought to determine the potential usefulness of RFB for MDR-TB patients in a high HIV prevalence setting. In our study population, 27% of MDR clinical isolates of *M. tuberculosis* retained sensitivity to RFB. This is in agreement with previous studies conducted in countries with relatively low HIV prevalence, including Turkey, Canada, Bangladesh, and Taiwan, where RFB susceptibility was found in 13–28% of RIF-resistant clinical isolates (Cavusoglu et al., 2004; van Ingen et al., 2011; Jamieson et al., 2014; Heysell et al., 2015). Moreover, the strong association, we observed between levels of resistance to RIF and RFB is consistent with other published reports (Sirgel et al., 2013; Jamieson et al., 2014). In our MDR isolate collection, the mutations S531L, H526Y, and H526D were primarily associated with resistance to both drugs, while D516V and L533P mutations were mainly associated with RFB-susceptible isolates. Similar MICs for both RIF and RFB have been reported for these mutations present among clinical *M. tuberculosis* isolates from Turkey, the Netherlands, and South Africa (Cavusoglu et al., 2004; van Ingen et al., 2011; Sirgel et al., 2013). In our study, RFB MICs for D516V isolates were significantly lower than those associated with the most common RRDR mutations (S531L and D526Y) and were consistent with RFB MICs reported elsewhere for this mutation (Cavusoglu et al., 2004; Jamieson et al., 2014). Moreover, no significant differences were found between RFB MICs of isolates with D516V or L533P mutations and those with wild type *rpoB* sequences. Interestingly, a double mutation at positions D516V-L533P was found in isolates with lower levels of RFB resistance. The treatment option may need to be investigated since increased RIFB concentration are not currently recommended due to possible toxicity issues (Sirgel et al., 2013).

All of the *rpoB* mutations identified in our study collection have been previously described and are reported in the TB Drug Resistance Mutation Database (Sandgren et al., 2009). Moreover, the mutations that, we found most frequently were similar to those reported from other surveys of *M. tuberculosis* clinical isolates. The S531L mutation is most often associated with RIF-resistance, ranging from 62 to 35% of clinical isolates surveyed (Cavusoglu et al., 2004; Campbell et al., 2011; Jamieson et al., 2014). Nonetheless, some variations in the relative proportions of specific *rpoB* mutations have been noted in different populations. For example, while we only found single representatives of S531R and S531W, these mutations were found to comprise up to 10% of clinical isolates in studies conducted in the United States and Turkey (Cavusoglu et al., 2004; Campbell et al., 2011). It is notable that all of the most common *rpoB* mutations seen in clinical isolates occur spontaneously in *in vitro* selected RIF-resistant strains of *M. tuberculosis*, indicating that the evolution of resistance is significantly constrained by structural requirements of the RNA polymerase (Morlock et al., 2000).

Current guidelines recommend RFB for treatment of TB patients with HIV co-infection or poor tolerance to RIF, only after safety and efficacy of the drug has been demonstrated (Nettles et al., 2004; Li et al., 2005; Horne et al., 2011). However, despite the known sterilizing properties of rifamycins, no randomized controlled trials have investigated the use of RFB in treatment of MDR-TB. The results of our study show that a proportion of MDR-TB cases may potentially benefit from the inclusion of RFB in chemotherapeutic regimens. Relevant clinical studies are needed to establish appropriate RFB-based regimens for achieving improved clinical outcome, particularly in HIV endemic regions. Our findings additionally support an association between specific *rpoB* mutations and RFB susceptibility, which can have implications for expanding therapeutic options in MDR-TB. For example, while *rpoB* mutations would not provide definitive classification of RFB susceptibility, molecular assays could be used as a basis for targeted susceptibility testing in MDR-TB patients.

The limitations of our study include the use of CLSI recommended clinical breakpoints for susceptibility testing of RFB (CLSI, 2011). However, a recent review and a study from a low HIV-TB prevalence setting have both suggested that the current breakpoint of 0.5 mg/L may be in need of revision (Schon et al., 2013; Crabol et al., 2016). In addition, as our study was focused on MDR-TB, our population of clinical isolates did not include any RIF-susceptible strains. Finally, as clinical data and patient demographics were not available for this study, we were not able to determine the HIV status of individual cases.

The study identified three novel mutations outside of the RRDR (V276L, V252E, and V276F) in RIF-resistant isolates, supporting inclusion of the 5′-end of *rpoB* in molecular testing for RIF/RFB resistance, as previously suggested (Tan et al., 2012). Moreover, the wide range of MICs seen in isolates with the same *rpoB* mutations suggests that other, as yet unidentified, genes may be contributing to the observed resistance levels. The presence of other genes that can confer resistance to rifamycins is also supported by our finding that 5% of clinical MDR isolates in the collection contained a wild type *rpoB* gene, two of which showed low level resistance to RBT. This observation is consistent with other studies, showing up to 5% of RIF-resistance in clinical isolates is not explained by mutations in *rpoB* (Ramaswamy and Musser, 1998; Sandgren et al., 2009) and by a recent report identifying a phosphotransferase in *M. tuberculosis* capable of inactivating RIF (Qi et al., 2016). The use of whole genome sequencing of clinical isolates of *M. tuberculosis* would be helpful in expanding our understanding molecular mechanisms of drug resistance.

## Author Contributions

IR, DF, and HS performed the experiments analyzed results, drafted the manuscript. AM and HM analyzed the data and edited the manuscript. GK, SO, HK, and AD edited and drafted the manuscript. NI conceived the study, analyzed results, and drafted manuscript. All authors read and approved the final manuscript.

## Conflict of Interest Statement

The authors declare that the research was conducted in the absence of any commercial or financial relationships that could be construed as a potential conflict of interest.
